# Voices from Pupil Participation in the Health Promotion Intervention “Pulse for Learning and Health [PuLH]” in Primary and Middle School

**DOI:** 10.3390/ijerph16224543

**Published:** 2019-11-17

**Authors:** Eva-Carin Lindgren, Katarina Haraldsson, Linn Håman

**Affiliations:** 1 School of Health and Welfare, Halmstad University, 30118 Halmstad, Sweden; linn.haman@hh.se; 2 Department of Food and Nutrition, and Sport Science, University of Gothenburg, 41120 Gothenburg, Sweden; 3 Department of Research and Development within Education, Region Jönköping County, 55111 Jönköping, Sweden; katarina.haraldsson@halmstad.se; 4 Department of Public Health and Community Medicine/Primary Health Care, Sahlgrenska Academy, University of Gothenburg, 41390 Gothenburg, Sweden

**Keywords:** children’s perspective, coaching, health promotion, learning, MVPA, pupils, well-being

## Abstract

In order to improve the learning conditions and health of schoolchildren, the Pulse for Learning and Health [PuLH] program in Sweden has introduced additional mandatory moderate to vigorous physical activity [MVPA] that lasts for 30 min three times a week. The PE teachers used a child-centered coaching approach to support all pupils. The aim of this study was to explore pupils’ perception and experience of PuLH that has been implemented in primary and middle schools in Sweden. We have taken into account children’s rights perspectives and adopted an exploratory and interpretive approach. In total, 73 pupils (34 girls, 39 boys, grades 4–9) were recruited through purposive sampling. 13 focus group interviews (*n* = 71) and individual interviews (*n* = 2) were carried out. All interviews were analyzed using qualitative content analysis. The analysis resulted in three major themes: ‘promotes academic performance and a learning school environment’, ‘promotes health and well-being’, and ‘individual and structural barriers’. From children’s perspective, the results highlight the importance of teachers and principals taking into account the interests and needs of all pupils, to have a well-planned MVPA intervention and to deal with issues regarding body ideals.

## 1. Introduction

There is evidence to suggest that moderate to vigorous physical activity (MVPA) has a beneficial effect on children’s and adolescents’ health [1]. However, the precise level of physical activity (PA) necessary to achieving different health benefits is currently unknown and is likely to vary across health outcomes [1,2,3]. Regarding the intensity level, evidence demonstrating that MVPA promotes more positive health effects than a lower level of PA [1,2,3,4] and dose-response relationships suggests that the higher the level of PA, the greater the health benefits [1]. Several PA recommendations have been published which state that children and adolescents (5–17 years) should engage in at least 60 min of daily MVPA [5,6,7]. This corresponds to approximately 10,000–11,700 steps per day among adolescents aged 12–19 years [8]. The recommendations also state that muscle/bone-strengthening should be incorporated ≥ 3 times per week [5].This level of activity has not been achieved for many children and adolescents [9]. Schools are considered to be an ideal arena for the promotion of regular PA among children and adolescents because this would mean that all children and young people would be reached. In Sweden, the time allocated to physical education and health (PEH) is two lessons (50–60 min each) per week in compulsory school (up to 15 to 16 years of age). PEH comprises three main parts: movement, health and lifestyle, and outdoor life and activities. However, according to school legislation, pupils should be offered the possibility of engaging in PA at school on a daily basis [10,11]. Scholars have stressed that the this legislation has not been implemented to any great extent [12]. Furthermore, because of the benefits of PA, the Swedish government has initiated plans to ensure that all children have a greater chance of becoming physically active [13].

Scholars have also suggested that participation in PA could have an immediate impact and long-term improvements on children’s cognitive and academic performance [14,15,16,17,18,19,20,21,22,23,24,25,26]. Scholars have also shown that participation in MVPA not only impacts academic performance, it also impacts social relationships and well-being [23]. Moreover, MVPA incorporates systematic training programs that are important for children to be able to improve, for example, their memory, as well as be capable of managing challenges both inside and outside the classroom [27]. However, it is not daily PA that has been shown to give this effect [27]. A number of previous studies have stressed that MVPA has either no beneficial impact or very limited beneficial impact on pupils’ executive control [28] and academic performance and sometimes even has a negative effect [29]. Other scholars have stressed that there is no convincing evidence to indicate either a strong or a significant relationship between PA and school performance [30]. To the best of our knowledge, only one study [31] has qualitatively examined pupils’ experiences of increased MVPA at school. However, the focus of that particular study was not on pupils’ experience of health, well-being and learning. In particular, there is a lack of knowledge about pupils’ views regarding additional MVPA in schools in respect of pupils’ perceived obstacles and opportunities to participate in MVPA, and the kind of influence MVPA may have from the pupils’ perspective. Furthermore, this study adds knowledge of a child-centered MVPA intervention in school, which is not run as part of the traditional curriculum. Listening to pupils’ voices regarding a child-centered MVPA intervention in school is crucial since their perception and experience could facilitate program development and improvement.

As not all children and young people are physically active during their leisure time and are therefore not used to physical exercise, it could be important to adopt children’s perspectives when pupils’ experience of MVPA are being examined. The United Nations Convention on the Rights of the Child (UNCRC) has outlined children’s rights. It stresses that children under the age of 18 are individuals who need special protection and assistance. The convention also stipulates that children have human dignity, the right to resources (e.g., health and education) and participation (e.g., being heard in all matters that affect them) [32,33]. In order to gain a picture of children’s needs, they must be given room to describe their experiences and feelings. Children’s right to voice their opinion in questions that concern them has also been emphasized [33]. Moreover, listening to pupils enables us to reflect on how the pupils experienced MVPA. Thus, the aim of this study was to explore pupils’ perception and experience of PuLH implemented in primary and middle schools in Sweden. More specifically, in order to allow pupils to be heard, we have focused on the following issues regarding PuLH: learning conditions and academic performance, health and well-being.

## 2. Materials and Methods

This study used a qualitative method based on an interpretive description [34] that acknowledges the constructed and contextual nature of human experience and allows for shared realities [35]. This interpretive position concerns the understanding of a phenomenon through an examination of the perception by its participants [36]. Thus, our interpretation of PuLH are seen from the pupils’ perspectives. Also, the authors of this study have a background in qualitative research methods and have solid knowledge of the literature on PA (interventions), health promotion, social-cultural perspectives on learning and UNCRC.

### 2.1. Planning for and Implementation of Moderate to Vigorous Physical Activity in Primary and Middle Schools

This intervention arose from schools’ need to increase pupils’ chances of improving their grades. Therefore, the planning for and implementation of MVPA in primary schools through the ‘Pulse for Learning and Health’ (PuLH) program started in eight municipalities (population 7000–30,000 inhabitants) in the region of Jönköping, Sweden, and involved eight primary and middle schools (from grades 4 to 9). *Stage 1*: Planning took place in the fall semester of 2016 and spring semester of 2017 in collaboration with researchers, PE teachers and development managers in each municipality in order to discuss the design of the intervention and research. Based on these discussions, the intervention was designed to promote health and well-being and improve learning conditions for the pupils. The design also incorporated a child-centred coaching approach, i.e., having a human rights perspective on children. This means creating an environment that is in the best interest of the child [37]. This approach allows children to play and enjoy themselves, is based on the specific child’s needs and circumstances and is therefore relative. The incorporation of a human rights perspective on children also fosters the development of friendships, encourages social interactions and enables children to express their own opinions (e.g., [38]). *Stage 2*: During the intervention, all relevant PE teachers (*n* = 22) at each school (approximately 18 per meeting) met the researchers in this study and other researchers in a collegial learning research circle [39] comprising three days of deep dive meetings. These meetings were arranged in order to increase knowledge of a coaching approach, discuss the process and implementation of the intervention, opportunities and barriers, as well as the design of the intervention, as mentioned above.

### 2.2. The PuHL Intervention

Three mandated aerobic exercise (MVPA) sessions were implemented, in addition to the two ordinary PE lessons. All pupils in this analysis participated three times/week for 6 months, except for illness. The PE curriculum (two weekly classes) remained unchanged during the intervention period. The exercise sessions lasted 30 min and during this time, the pupils were supposed to be in a heart rate zone between 60% and 80% of their maximum heart rate for at least 20 min. In order to monitor their heart rate zones, the pupils wore a pulse sensor (Polar or Activio) connected to a monitor. All pupils were subject to a maximum heart rate test before the intervention started. This was recommended by a researcher in physiology. The MVPA sessions in PuLH were supervised by the PE teachers in charge and other school staff. The activities comprised different games, non-competitive sporting activities, dance, workouts, indoor cycling, etc. Adapted MVPA was offered to pupils with neurodevelopmental disabilities (NDD) and physical disabilities, if needed.

### 2.3. Recruitment and Participants

This study mainly relied on focus group interviews (FGIs) but also individual interviews in order to produce empirical data as this allowed the pupils to express themselves freely and be heard [40,41]. Thirteen FGIs and two individual interviews comprising 73 pupils (34 girls, 39 boys, grades 4–9) were conducted after six months, during the spring semester 2018 at the intervention schools (Table 1).

**Figure 1:**
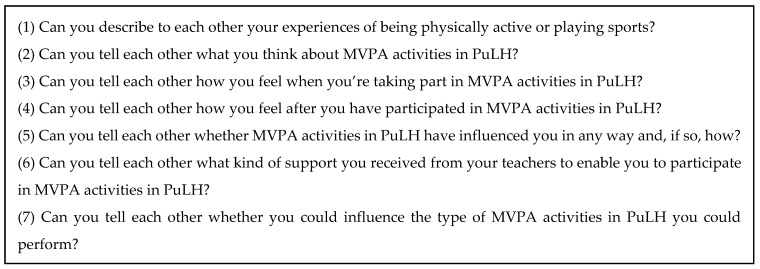
Interview guide.

One of the FGIs was a triad (three pupils) and the other FGIs included five to seven pupils (cf. [42]). The pupils were recruited purposively. The purposive sample was based on (a) different grades in the intervention schools, (b) gender, (c) diversity regarding pupils’ level of experience of PA and sport. Moreover, we placed pupils who had similar backgrounds in the same FGIs because such groups could provide a safe environment in which pupils felt comfortable sharing their views and feelings without being ridiculed [43]. Thus, FGIs were conducted with homogeneous groups of boys and girls who were identified by their PE teachers as being either generally ‘active’ or more or less ‘inactive’ in their leisure time and were in a similar age group. All schools were located in mixed socioeconomic areas. All interviews (FGIs and individual interviews) were conducted at the pupils’ schools and we attempted to develop a relationship of trust between the pupils and ourselves as interviewers. The interviews lasted between 15 and 84 min with an average duration of approximately 39 min. Interviews were chosen in order to produce data, since pupils themselves are supposedly the best source of knowledge regarding their own experiences of participating in the PuLH intervention (cf. [41]). In all interviews (FGIs and individual interviews), the first or last researcher acted as a moderator. An interview guide (Figure 1) was developed and tested in two FGIs conducted by the first and last author in order to capture details regarding the pupils’ perceptions. While this pilot FGs did not alter the core questions of the interview guide work, follow-up probes were modified to allow for detailed discussion of these specific factors. Thus, these two pilot FGs were included in this study. All interviews were audio recorded and transcribed verbatim.

### 2.4. Ethical Considerations

The pupils and their parents were informed about the objective and nature of the study and we asked parents if their children were permitted to participate in the study. As the pupils were under 18 years of age, both parents and pupils were informed about the study and were asked to provide written consent to their participation. The presentation of the study stated that the pupils’ participation was voluntary, that they could withdraw at any time, and that any information provided would be treated confidentially. All parents/pupils who were contacted agreed to participate in the study. To ensure confidentiality, pupils have been given pseudonyms. The study was approved by the Regional Ethical Review Board of Lund University (DNR 217/601). The empirical data was decoded and information about the pupils was processed separately. Only the study researchers had access to this information. Guidelines regarding data are based on the Swedish Archives Act (SFS1990:782) [44] and information about the pupils is subject to the General Data Protection Regulation (GDPR2016/679) [45].

### 2.5. Data Analysis

Qualitative content analysis [46,47] was considered suitable for thematizing the data. The data analysis process involved six steps. While this analytical process may resemble a linear process, it actually involved going back and forth between decontextualization and contextualization which, although complex, creates analytical depth. First, all interviews were transcribed verbatim and the transcripts were read multiple times in order to gain an overall impression and a general understanding of the pupils’ perspectives. Second, the meaning units were marked and condensed. Third, the meaning units were coded. Fourth, in order arrange the codes into tentative subthemes, similarities and differences between the codes were sorted and compared. Fifth, the tentative subthemes were discussed and reviewed multiple times by the researchers until they were encoded into 13 subthemes. Sixth, adhering to children’s perspective, the researchers moved back and forth between the data and literature in order to interpret the pupils’ perception and experience about the PuLH program. In this step, the subthemes were arranged into three main themes (see Table 2 for an example of the data analysis from meaning units to themes). Although the first and last authors performed the analysis, all steps were regularly discussed among all members of the research group in order to reach a consensus. Quotations from pupils were chosen to illustrate the themes. These are discussed below.

## 3. Results

### 3.1. Promotes Cognitive Performance and a Learning School Environment

**Figure 2:**
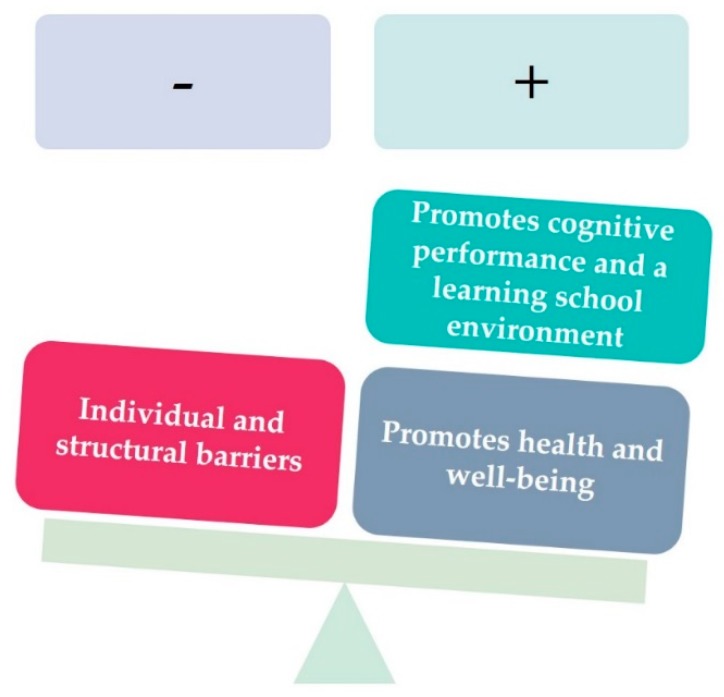
Empirical model summarizes the study’s findings, based on the pupils’ voices.

The empirical model summarizes the study’s findings based on the pupils’ voices and contains three main themes: (1) Promotes cognitive performance and a learning school environment; (2) Promotes health and well-being and illustrates pupils’ positive experiences of PuLH; (3) Individual and structural barriers illustrate pupils’ negative experiences of PuLH (Figure 2).

#### 3.1.1. Improved Memory, Concentration, and Motivation

Most pupils, except for pupils who frequently participated in organized sport or physical activity in their leisure time, stated that MVPA in PuLH had influenced their memory, concentration and motivation. They stated that they had become more alert in lessons immediately after MVPA. They also stated that they could think better and faster and that their memory had increased after MVPA in PuLH, as one of the pupils said: “For example, you remember more, and school is much simpler” (FGI 5). Furthermore, they stated that their concentration and motivation to study increased and that they could understand more of the teaching immediately after the MVPA. “I feel like I’m more focused… and this means I maybe understand more of the lesson and what my teacher is talking about. If I understand more, it’s more fun, and I feel more motivated to study” (FGI 12). This is consistent with previous research highlighting that MVPA improves pupils’ cognitive performance [11,12,13,14,15,16,17,18]. Additionally, some of the pupils who were non-physically active in their leisure time stated that MVPA in PuLH positively influenced their capacity to do homework. “… you think better afterwards when you do your lessons and stuff” (FGI 3).

Furthermore, some of the pupils explained that they had NDD or were hyperactive and that MVPA in PuLH freed up their energy and helped them regain their attention. One of the boys stated that MVPA had made a huge impact on his life and that it had been a decisive factor in helping him manage his schoolwork.

Moderator: Do you remember what school was like before you had PuLH?

Lucas: Hm… it was really bad.

Moderator: It was bad?

Lucas: Yes.

Moderator: In what way was it bad?

Lucas: I couldn’t concentrate. I didn’t do my assignments or anything at school.

Moderator: What if you didn’t have PuLH in school…

Lucas: Hm.

Moderator: What do you think about this?

Lucas: I probably wouldn’t have been able to do anything in class if we didn’t have PuLH.

(I 1)

In a similar vein, a recent review has shown the positive effects of PA on the cognition of young people with NDD. However, the review also showed that the benefits of PA differ according to the intervention time and the duration of the effects on behavior can be contradictory and vary depending on age [48].

However, some of the pupils who frequently participated in organized sport or physical activity in their leisure time stated that MVPA in PuLH had no influence on their memory, concentration and motivation and felt they had been manipulated into believing that participation in MVPA would improve their grades.

#### 3.1.2. A Calm and Peaceful Classroom

In general, MVPA in PuLH was described by the pupil’s as having a calming effect on themselves or their classmates and created a more peaceful classroom environment, which could be beneficial for their learning conditions. The pupils stated that it was less noisy and quieter in the classroom with specific regard to MVPA since they or their classmates managed to focus more in the classroom after MVPA in PuLH. One pupil talked about how MVPA had affected the class:

“In my class, I’m in B, and they’re in A, anyway… in my class, there are a few guys who swore a lot… who couldn’t sit still in class … who stood on the table and shouted … and now, they’re calmer and they sit in their chairs and … we’re really quiet”.

(FGI 6)

Some pupils also stated that they were lesser hyperactive and restless at school and home after MVPA in PuLH started at school. In the following excerpt, one pupil stated that his surplus energy decreased after MVPA and described this calming effect as follows: “and your brain doesn’t feel tired, only your legs, so that’s really only okay. I’m not actually able to move around afterwards, so I can’t disrupt (the class anymore), ha ha ha.” (FGI 12). This statement reinforces the idea that MVPA could contribute to a peaceful classroom, which is also described in Chaddock et al. [27].

### 3.2. Promotes Health and Well-Being

#### 3.2.1. Healthier Living Habits and Improved Health Outcomes

In terms of healthy habits, the non-physically active pupils (in their leisure time) stated that MVPA in PuLH had influenced and inspired them to start exercising in their leisure time and to eat healthier food. For example, they stated that by trying different forms of physical activities, they realized that it could be enjoyable and not too difficult. They also stated that they were motivated to eat healthier foods more regularly, as shown in the following excerpt. “But, I sort of feel like this, if I’m going to exercise every day, I might as well just eat well every day” (FGI 4). Also, non-physically active pupils noted that they slept better, had reduced exercise-induced asthma, experienced less pain in their backs, shoulders, stomachs, as well as less headaches after starting MVPA. One of the pupils stated. “I’ve had headaches, although, since we started (with PuLH), I haven’t felt that bad, and so I feel better, and I’m not as tired at school anymore” (FGI 5). The findings that MVPA in PuLH has beneficial effects on pupils’ health are in accordance with previous studies [1,3,49].

#### 3.2.2. More Alert and in a Better Mood

Most of the participants, despite the pupils who frequently participated in organized sport or physical activity in their leisure time and some of those who did not receive refreshments, stated that they felt more alert and in a better mood after MVPA in PuLH. A better mood refers to how pupils stated that they felt they were lesser grumpy, angry, irritated and lazy and were happier, and that they felt good during both the activities and after MVPA in particular. One of the pupils stated that MVPA affected their mood: “And you notice that people are in a better mood when they’ve been to the PuLH activities, they’re much happier, and if they weren’t there, you’ll notice that then you notice that … some of them can be grumpy and a little bitter” (FGI 9). A recent systematic review shows that MVPA, both directly and indirectly, was associated with mental well-being and quality of life in adolescents [3,4].

#### 3.2.3. Needs, Interest, and Participation in Focus

Most of the pupils’ accounts revealed that the teacher let them talk about their needs and interests regarding meaningful and enjoyable MVPA in PuLH in which they wanted to participate. According to the pupils, regardless of their background, gender and skills, everyone had the opportunity to influence and decide what activities they liked doing. In the following excerpt, we can discern that it was important for pupils to gain influence over the activities because they could then choose activities that they perceived as being enjoyable.

Moderator: Were you able to participate and decide on what you wanted to do at the PuLH class?

[Several respond “Yes” in different ways, all speaking at the same time.]

Moderator: What does it feel like when you can decide on these activities yourselves?

Lynn: Yeah, it’s fun.

Moderator: Hm.

Lynn: That you can also decide a little yourself…

[Several say “Yes.”].

(FGI 1)

The fact that the pupils felt they were being heard and had the opportunity to influence and decide the content of the activities contributed to a positive experience [50]. It could increase pupils’ PA [51] and affect the ability of physically non-active pupils to find a PA in which they wanted to participate, even in their leisure time. Most of the pupils, except some of the boys who frequent participate in organized sport or physical activity in their leisure time, stated that they felt the activities in PuHL were pleasant, refreshing and enjoyable, which also was important for them. These findings are consistent with previous studies which have highlighted the need for adolescents to engage in MVPA or PA that they find personally meaningful and enjoyable [52,53,54,55].

They also stated that the activities gave them space for recreation and recovery in which they didn’t need to worry about rating and assessment. One of the pupils said. “It’s great to just let go of school work and not need to think about what you’re going to do and how you’re going to perform, but just have fun and compete with yourself” (FGI 12). However, boys who frequently participated in organized sport or physical activity in their leisure time were dissatisfied that their heart rate was only allowed to achieve 80% of their maximum heart rate during the activities.

When the pupils are heard it can be regarded as part of a participatory approach in which pupils, irrespective of gender, skills, ethnicity, socioeconomic area or disability had equal opportunities and the right to determine what they could do in the PuHL. This complies with the UNCRC perspective [32]. Also, a number of girls stated that it was good that they had the opportunity to influence the content of PuLH, but that they felt embarrassed because they were afraid that their choices would not be appreciated by their classmates, as shown in the following excerpt:

Sara: [That choosing an activity is] both fun and a little embarrassing, because you don’t know what the others like doing.

Jessica: Nope, because maybe you stand there at the front and you say these are the games we’re going to play, and then they just moan and say, like, “no, that’s boring” or whatever…

Moderator: Hm… well, how do you think you should choose an activity?

Jessica: Um, like what would they like, and what do we think is fun and stuff like that….

(FGI 1)

This could be the result of gender norms and ideals, which can create dominant symbols and a belief system that influences the thoughts of certain girls regarding what they are allowed to decide [56].

#### 3.2.4. Satisfied with Body Shape and Increased Physical Fitness

By participating in MVPA sessions in PuLH, the non-physically active pupils stated that their physical strength and fitness had developed, which influenced their level of satisfaction with their body and their well-being:

Peter: They have changed my life.

Robin: Yeah, it has, like our bodies have changed a lot and we see the difference now and so we’re happy, and we’re not like this… I feel bad because my body is bad… that’s the way it is, right… so you notice and then you feel better as a person.

(FGI 7)

They also stated that they had become less lazy and more aware of their improvement, both bodily and physically. This was particularly evident in pupils who were physically inactive in their leisure time and/or behaved in a sedentary manner. One of them said: “In the seventh grade, I couldn’t walk half a kilometer without getting breathless… but now I’ve noticed a really big difference. I had a very low stamina level before” (FGI 7). These findings are in line with a systematic review and meta-analysis that have identified that PA was strongly associated with perceived physical self-concept, which includes perceived competence, perceived fitness and perceived appearance [57].

#### 3.2.5. Cohesion and New Friends

The pupils stated that the MVPA in PuLH increased cohesion within the classes since the activities were often based on team building. They appreciated collaborating with classmates or other pupils they usually didn’t socialize with because they made new friends and felt an increased sense of social interaction.

Moderator: You said that there was better cohesion in the class…

Stella: Yes, much better, my seventh grade class was in a state of chaos. There were gangs here and gangs there but now there is more cohesion.

Moderator: Do you think PuLH has anything to do with this?

Stella: Yes, we do a lot of exercises in cooperation, or a lot of team sports and games, and then you end up talking to people who you wouldn’t usually talk to…

Moderator: Who you wouldn’t normally…

Stella: Who you hadn’t hung out with before.

(FGI 12)

As Wentzel and Ramani [58] pointed out, it is important to promote social relationships with fellow pupils because they can impact pupils’ mental well-being.

### 3.3. Individual and Structural Barriers

#### 3.3.1. Uncomfortable Showing Their Bodies

The pupil’s statements regarding participating in MVPA in PuLH showed that the non-physically active girls in particular were uncomfortable about exposing their bodies in the locker room, in the shower, as well as during activities. They stated that they were dissatisfied with their appearance and body shape and because they felt they had unattractive body shapes they were unwilling to participate in MVPA in PuLH. Furthermore, the pupils also stated that they didn’t want to expose their bodies because they did not possess the sufficient competence, capacity and/or skills to perform certain MVPA activities. The following excerpt highlights a body-focused discourse specifically about girls negotiating body ideals and lack of skills:

Julia: Sometimes, I can’t participate because I hate showing my body, and that’s just the way it is, and I hate my gym clothes, I hate having to change clothes and there’s many reasons why I don’t participate sometimes. It’s not because of makeup, but just because I hate showing my body both to my very best friend and to someone I hardly know.

Moderator: You don’t like the activity, itself, or what?

Julia: Yeah, but not changing in the locker room and showing my body like that.

Thelma: In tight clothes.

Moa: That’s kind of what it’s about, and it’s about if you don’t know how to do something and don’t want to participate because of that.

Julia: It’s not about makeup.

(FGI 9)

In a similar vein, pupils in Wiker’s study on PEH [59] stated the same thing, i.e., that they didn’t want to expose their bodies in showers and locker rooms and therefore didn’t always feel safe and physically protected.

#### 3.3.2. Poor Planning

The pupils at some of the schools stated that their school had not a well-planned school schedule to ensure sufficient time between lessons and MVPA in PuLH. This issue affected the students to experience of stress.

The pupils also stressed that it was important to have MVPA in the morning in order to benefit from the activities afterwards. A number of schools had implemented MVPA at other times, for example, before lunch or at the end of the school day. The pupils in grades 7–9 emphasized the importance of adequate lunch planning at the school in order to benefit from MVPA in PuLH. However, this was specific to boys who engaged in many sports in their leisure time. Otherwise, they became tired and negative, as expressed in the following excerpt:

John: I can’t function afterwards (after PuLH) when I’m hungry…

Jacob: Sometimes, the amount of food you get is rather limited. That’s tough.

John: When you’re still hungry, you can’t take more, and that’s tough.

(FGI 10)

It is important that the pupils receive a sufficient amount of food at lunch in order to manage their schoolwork, especially if they have MVPA three times a week. It was the pupils at schools that offered refreshments after MVPA in particular who were generally satisfied with PuLH. In grades 7–9 at schools that did not offer refreshments after MVPA, pupils complained because they felt a lack of energy.

Furthermore, in all FGIs, the pupils stated that nearly all of them participated in MVPA in PuLH each time. However, some of the girls did not. Because of this, both girls and boys emphasized that teachers should try and include all girls in MVPA. The pupils who had colds or who not wearing suitable clothing did not participate in MVPA in PuLH and were therefore told to go for a walk or sit on the bench. This is consistent with a report by the Swedish School Inspectorate [11] which shows that PEH teachers often exclude pupils from PEH lessons if they are not wearing suitable clothing.

#### 3.3.3. Problems with the Heart Rate Monitor

In some of the focus groups, in all grades and among both genders, the pupils stated that it was difficult to achieve the “right” pulse zone and be in the “right” pulse zone for at least 20 min. This applied to pupils whose pulses were either too high or too low. These pupils stated that this was frustrating or that they considered it as a failure. The pupils also stated that it was difficult to have an overview of both the monitor and the activities in which they were participating, as can be seen in the following excerpt:

Clara: I actually thought that if someone [a student] chooses something that is a little too fast, so that you are red or orange [too high a pulse rate] easy… it’s not easy to keep track of your pulse… when you have to run around so that no one catches you.

Moderator: Right! Ah, that can be hard.

Clara: Because when I tried to check my pulse, sometimes I got caught nearly every time we played.

[Many laugh]

Moderator: What did you think then?

Wilma: Um, that there are some [students] who grab you and push as hard as they can when you’re checking your pulse, and that hurts quite a bit.

(FGI 5)

The requirement to have a sufficiently high pulse rate for 20 min while adhering to the game rules and interacting with their classmates can be both complex and challenging for the pupils.

## 4. Discussion

This qualitative study shows pupils’ perception and experience of PuLH, based on a child-centered coaching approach implemented in primary and middle schools in Sweden. One of the main findings indicates that several pupils stated that PuLH influenced their memory, concentration and motivation to study. However, the pupils who frequently participated in organized sport or physical activity in their leisure time did not express this view but stated that PuLH had positively impacted their classmates. A recent review has shown strong evidence of a positive association between cardiorespiratory fitness and physical fitness with academic performance in cross-sectional studies, as well as evidence of longitudinal studies showing a positive association between a cluster of physical fitness and academic performance. However, barely one third of the studies meet the criteria for estimating statistical power [60]. The results of a study by Quinto Romani and Klausen [29] using data from a randomized school-based intervention indicate that, on average, the studied intervention of 45 min extra PA during the school day has a minimal beneficial impact on the pupils’ academic performance and, in some cases, even a negative impact. Pupils also stated that PuLH contributed to creating a calm and peaceful classroom environment and that it was even decisive for pupils who had been diagnosed with NDD. The universal health promotion work of Swedish schools highlights the importance of creating a secure and peaceful learning environment for pupils [61]. It is possible that MVPA could be a means of achieving this. Our interpretation is that PuLH has been valuable for pupils’ learning conditions although it cannot be claimed that PuLH contributes to learning or improved grades. Thus, is it important that teachers do not communicate the idea that MVPA is a panacea. This is important because; (a) research has been unable to prove that MVPA affects academic performance, (b) it is not clear what level of intensity and duration of MVPA impacts pupils’ academic performance, (c) there is a risk that schools will use MVPA activities as a quick fix, i.e., that MVPA activities are expected to solve every complex problem regarding pupils’ academic performance, (d) if teachers do not listen to their pupils, those pupils who have little or bad experience of MVPA activities and sports may not continue to participate. This could lead to continued health inequalities between the genders since more girls are non-physically active in their leisure time.

Another main finding is that most of the pupils stated that MVPA in PuLH was important to them as it improved their sense of well-being. According to WHO’s [62] definition of health. well-being is an important dimension and is partially accounted by an individual’s subjective experience. The pupils stated that they felt in a better mood and lesser grumpy, angry and irritated after MVPA in PuLH. This could be partially related to Keyes’ [63] description of emotional well-being and Diener’s [64] description of the relative incidence of positive emotions (pleasant affect) and the relative absence of negative emotions (unpleasant affect). However, the pupils who frequently participated in organized sport or physical activity in their leisure time did not state that they felt an improved sense of well-being after MVPA in PuLH. One critical question is whether MVPA should be directed at high-activity sporting pupils who regard an additional MVPA as being superfluous. Another critical question is whether MVPA should be directed at individual pupils with greater needs such as non-physically active pupils and pupils with NDD. Even pupils who had been offered a sufficient lunch and refreshments did not indicate that they felt an improved sense of well-being after MVPA in PuLH. Since we already know that few pupils meet the current recommendations concerning dietary intake [65,66], it is even more important that pupils are offered a sufficient amount of food at school when MVPA is implemented.

Moreover, pupils who were non-physically active before the PuLH program stated that MVPA had influenced and inspired them to adopt healthier lifestyle. When teachers listen to the views of pupils about their needs and interest in MVPA, it could help pupils change their habits and further engagements. However, some of the pupils, especially girls, did not participate in MVPA in PuLH every time. This is a challenge for teachers, and it is important to promote MVPA through a respectful and helpful approach and ensure that the views of non-physically active pupils (girls) are taken into account. When teachers give pupils the opportunity to engage in interest-based activities, it can strengthen their belief in themselves which, in turn, can increase their motivation to become involved. Placing pupils at the center of this study also means letting all pupils, regardless of gender or level of experience of PA and sports, participate in decision-making regarding the choice of activities. This was generally appreciated by the pupils in the study. Allowing pupils to take part in decision-making could influence their subjective experiences of health (e.g., [67]), knowledge acquisition and skills [68] and can be regarded as a form of empowerment [50].

However, it is important that teachers reflect on how to build a social and trusting climate in their classes so that pupils are brave enough to choose activities in front of their peers and not feel embarrassed. PuLH can be seen as an example of health-promoting work in school that [61] to strengthen and maintain pupils’ health and well-being.

Furthermore, on the one hand, the findings show that, prior to the PuLH program, the non-physically active pupils stated that they had become more satisfied with their body shape and had increased their physical fitness during the PuLH program. On the other hand, those pupils who still were non-physically active, especially girls, continued to feel uncomfortable about showing their bodies. This individual barrier might particularly risk undermining girls’ willingness to engage in MVPA and therefore exclude them from participating, which is similar to the findings of other scholars [55,69]. In order to teach pupils to have a more accepting attitude to different kinds of body appearance, PE teachers should initiate discussions from a norm-critical perspective, thereby initiating reflective dialogue among pupils about perceptions in society and body ideals [70,71].

One of the structural barriers that concerned the pupils was insufficient transition time between the PuLH session and their next lesson. Sometimes pupils were reprimanded by teachers because they were late to class. In some ways this is surprising given that PuLH was a common strategy in all participating schools. When planning for an MVPA intervention, it is important to have a solid plan for such an intervention to ensure that it does not adversely affect pupils. Another structural barrier concerns problems with the pulse monitor. Some pupils had problems achieving the “right” pulse zone and having an overview of the activity and the monitor simultaneously. Since it is difficult to measure the maximum heart rate for children and adolescents in schools, we would question whether this type of digital technology is necessary when implementing MVPA in schools. Perhaps the Borg Scale (ratings of perceived exertion) would be sufficient [72] so that pupils can assess and specify their level of exertion.

As for methodological limitations, our findings are based on a small sample of Swedish children (*n* = 73) and these do not completely reflect the diversity of the population in Sweden. Therefore, the findings can only be transferred to similar groups of pupils. However, it was not our intention to generalize the findings from this qualitative study. Another limitation was that the excerpt, which was translated by an English language expert, was not back translated into Swedish. Nor has there been any cross-checking with the pupils of the interpretation of their perception and experience to ensure that the true meaning was reflected. However, we described the interpretation of the preliminary findings with the teachers in the research circle in order to discuss whether or not they recognized the findings and also provide feedback for future work with PuLH. A strength of the study was that, as a research team together with the teachers who ran PuLH, we had a collegial learning research circle during the intervention in order to engage in a continuous dialogue about the quality of the implementation of PuLH. In these meetings, we discussed how the process of the intervention should proceed, possibilities and barriers and how to handle any obstacles.

## 5. Conclusions and Implications

This study is novel in that it provides insights into pupils’ perception and experience of an MVPA intervention at school based on a child-centered coaching approach that is not run as part of the traditional curriculum. It is reasonable for MVPA activity to be introduced within a school’s framework, primarily with regard to health benefits and pupils’ well-being, but also because pupils’ learning conditions can be improved. It is likely that learning conditions will be improved when the class becomes calmer and when pupils realize that they are able to concentrate more after MVPA. Not least, PuLH appears to have been particularly favorable for pupils who are non-physically active in their leisure time and for pupils who have NDD.

The results also stress the importance of taking the interests and needs of all pupils into consideration in order to achieve a well-planned MVPA intervention and also the importance of planning how to deal with issues regarding body ideals.

Moreover, it may be necessary for schools and teachers to include pupils in designing interventions to ensure they meet the needs of all pupils and provide good conditions and opportunities for them to participate in MVPA that is enjoyable and does not generate a feeling of stress before or after MVPA activities. A potential direction for future research could be to observe MVPA sessions in order to gain further insight into children’s learning, health and well-being, as well as gain a critical understanding of the workings of power, privilege and subordination of children.

## Figures and Tables

**Table 1:** Key characteristic of the pupils.

Characteristics	Girls	Boys
Grades 4–6	18	20
Grades 7–9	16	19
Frequently participate in sports in leisure time	8	5
Participate in sports or PA in leisure time	13	23
Non-physically active in leisure time	13	11

**Table 2:** Examples of meaning units, codes, subthemes, and themes from the data analysis.

Meaning Unit	Code	Subtheme	Theme
Moderator: What does it feel like in class after you began with activities that increased your pulse rate (MVPA in PuLH)? Carl: Actually better. Timmy: Hm, you’re more focused. Carl: Right, and can sit still more (FGI 2)	Focused Sit still	A calm and peaceful classroom	Promotes cognitive performance and a learning school environment
Thomas: before, I hated running and stuff like that but now I’ve begun to like it because I know that it’ll make me feel good afterwards (FGI 11)	Begin running Feel good	Healthier living habits and improved health outcomes	Promotes health and well-being
Mary: You don’t always get 10 min because sometimes they run a bit late so that you have less time (to change) Moderator: Right. Susan: Really, 10 min isn’t enough time, because first of all, you have to change and shower, and stuff like that. Then you have to get dressed, then you go and turn in your things, phone and keys and then it gets a bit stressful because you have a lot to do, and 10 min isn’t enough time (FGI 5).	Stressed for time afterwards	Poor planning	Individual and structural barriers
